# Stepwise intensification of insulin therapy in Type 2 diabetes management—exploring the concept of the basal-plus approach in clinical practice

**DOI:** 10.1111/dme.12019

**Published:** 2013-02-20

**Authors:** D R Owens

**Affiliations:** Institute of Molecular and Experimental Medicine, Cardiff University, University Hospital of WalesCardiff, UK

## Abstract

Basal insulin provides an effective method for initiating insulin therapy in people with Type 2 diabetes, resulting in significant improvements in glycaemic control, lower rates of hypoglycaemia and less weight gain than either prandial or premixed insulin regimens. However, the progressive nature of Type 2 diabetes and the inability of basal insulin to correct excessive postprandial glucose excursions mean that insulin therapy will eventually need to be intensified, typically by adding prandial insulin as part of a basal–bolus or premixed insulin regimen. The aim of this review is to summarize recent clinical evidence for a staged ‘basal-plus’ strategy for insulin intensification where one, two or three prandial insulin injections are added to basal insulin according to individual need. In the early stages of insulin therapy, most individuals seem to achieve favourable glycaemic control with basal insulin alone, or in combination with a single prandial insulin injection. The addition of a single prandial insulin injection at the largest meal is well tolerated and associated with significant improvements in glycated haemoglobin (HbA_1c_), low rates of hypoglycaemia and limited weight gain. More people achieve recommended HbA_1c_ targets with a basal-plus strategy, compared with twice-daily premixed insulin therapy, with lower rates of hypoglycaemia. The data indicate that a step-by-step approach with the basal-plus strategy is a promising alternative method of insulin intensification that allows for individualization of treatment and may delay progression to a full basal–bolus insulin replacement therapy for many individuals.

## Introduction

The relentless, progressive nature of Type 2 diabetes results in an almost inevitable need for insulin supplementation and its intensification in an attempt to combat a worsening glycaemic profile 1,2, including glycaemic variability 3 and the associated increased risk of vascular complications 4–7. Deteriorating glycaemic control with disease progression is now understood to follow a sequence from an initial inadequacy in prandial glycaemic control through to the addition of fasting hyperglycaemia. This process usually begins with excess postprandial hyperglycaemia during daytime, followed by fasting hyperglycaemia because of increasing hepatic glucose production overnight, culminating in sustained hyperglycaemia 3. Even in the presence of ‘apparently good’ glycaemic control, overexposure to postprandial glucose excursions 8,9 and its consequences persists 3,4,10–12.

The American Diabetes Association (ADA)/European Association for the Study of Diabetes (EASD) guidelines propose that a glycated haemoglobin (HbA_1c_) level of ≥ 53 mmol/mol (≥ 7%) should ‘serve as a call to action’ to initiate or change therapy with the goal of achieving a level below 53 mmol/mol (7%), although it is recognized that a patient-centred approach should be adopted, with clinical judgement used to allow some flexibility in the application of this target, taking into account each individual's clinical characteristics (e.g. presence of cardiovascular disease), circumstances and preferences 13–15. Even with optimal use of basal insulin, it is estimated that, using current treatment paradigms, ∼40% of people will not meet HbA_1c_ recommendations of < 53 mmol/mol (< 7%) 16,17. In the face of rapidly rising diabetes prevalence, it is expected that insulin therapy will increasingly be undertaken in primary care 18 and, as such, it is vital that physicians are fully aware of the available options to allow them to select the optimal regimen for each individual with Type 2 diabetes. A well-considered step-by-step approach to the intensification of insulin therapy, adding one, two or three prandial insulin injections to basal insulin according to each individual's prandial requirements, seems a logical way forward. This review will explore the current evidence underlying this concept of meal-driven insulin intensification for the treatment of Type 2 diabetes, as well as the implications of adopting such an approach in clinical practice.

## Current recommendations and insulin therapy options

**FIGURE 1:**
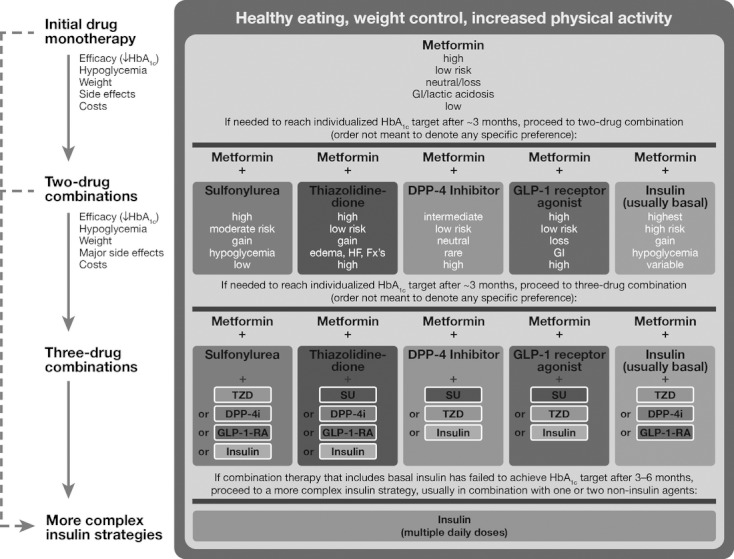
American Diabetes Association/European Association for the Study of Diabetes recommendations for the treatment of Type 2 diabetes 19. DPP-4, dipeptidyl-peptidase-4; Fx's, fractures; GI, gastrointestinal; GLP-1, glucagon-like peptide 1; GLP-1-RA, GLP-1 receptor agonist; HF, heart failure; SU, sulphonylurea; TZD, thiazolidinedione. Reproduced from Inzucchi *et al*. (2012) with permission from the American Diabetes Association.

The current ADA/EASD guidelines recommend that individuals with Type 2 diabetes are initially treated using lifestyle modifications; once this fails to maintain HbA_1c_ levels < 7%, they should be progressed to metformin monotherapy (Fig. 1) 19. If up to 3 months of metformin monotherapy does not enable the individual to reach the HbA_1c_ target, they should be progressed to a two-drug combination of metformin with a sulphonylurea, thiazolidinedione, dipeptidyl peptidase-4 inhibitor, glucagon-like peptide-1–receptor agonist or basal insulin. If after 3 months the HbA_1c_ remains above the glycaemic target set for the individual, therapy should then be progressed as represented in Fig. 1 19.

**FIGURE 2:**
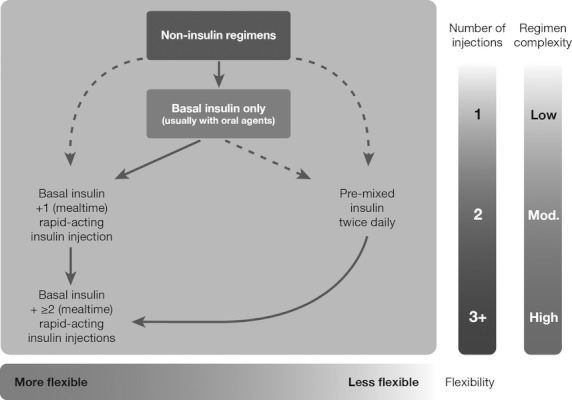
American Diabetes Association/European Association for the Study of Diabetes recommendations for the sequential intensification of insulin therapy 19. Reproduced from Inzucchi *et al*. (2012) with permission from the American Diabetes Association.

When a three-drug combination therapy with basal insulin does not achieve HbA_1c_ targets, then a more complex insulin strategy is recommended. The two possible strategies are (1) to continue with the basal insulin and add rapid-acting insulin in a stepwise manner or (2) to transfer to twice-daily premixed insulin (Fig. 2). The more flexible stepwise addition of prandial insulin to basal insulin is the preferred strategy from the guidelines 19. Eventually, it may be necessary to progress to a full basal–bolus regimen to achieve the desired glycaemic target.

The stepwise addition of prandial insulin has been investigated in several clinical trials. The addition of a single prandial insulin injection to the existing basal regimen before breakfast or the main meal, or before the meal consistently with the highest postprandial glucose, is referred to as a ‘basal-plus’ strategy 20,21. This basal-plus strategy has been identified as effective when intensifying insulin therapy, before a full basal–bolus regimen is considered 22.

### Basal insulin therapy

Insulin is acknowledged as being the most consistently effective therapy for lowering blood glucose. The Treat-to-Target Trial (4-T) demonstrated the benefit of initiating insulin therapy with basal insulin in persons with Type 2 diabetes inadequately controlled by oral hypoglycaemic agents, compared with prandial or premixed insulins 16. After 1 year, 41.7, 27.8 and 48.7% of people reached HbA_1c_ ≤ 53 mmol/mol (≤ 7.0%) in the biphasic, basal and prandial groups, respectively 18, and after 3 years 49.4, 63.2 (*P* = 0.02 vs. biphasic) and 67.4% (*P* < 0.001 vs. biphasic) reached this target 17. People initiating insulin treatment with basal insulin experienced significantly lower rates of hypoglycaemia and less weight gain compared with prandial and premixed insulin regimens. Thus, this study provided the first clear evidence in favour of starting insulin-based treatment with basal insulin. The study also showed that, over the longer term (3 years), a premixed insulin regimen with a midday prandial bolus (which could be added if required) was not as effective as basal plus prandial insulin at attaining and maintaining treatment targets 17,18.

## Basal-plus: basal insulin plus prandial insulin one step at a time

Having titrated the basal insulin against the fasting glucose, the inability to achieve glycaemic control despite normal or near-normal fasting glucose usually means that excessive glycaemic excursions may be occurring during the day, following either breakfast or the main evening meal. Therefore, a prandial insulin injection before the meal most consistently contributing to the greatest postprandial glycaemic excursions is a logical first step in progressing insulin therapy, thereby allowing for a more gradual intensification of insulin therapy governed by structured self-monitoring of blood glucose 23. Adjustments in the doses of both the basal and prandial insulin may be required during this process. Additional injections of prandial insulin may eventually be required, leading to a full basal–bolus regimen based on disease progression 23. This strategy is recommended by the ADA/EASD consensus guidelines 19.

The alternative to a basal-plus strategy is to use a premixed insulin analogue regimen, previously mentioned in the Treat-to-Target trial discussed earlier. Premixed insulin analogues combine a fixed ratio of basal and prandial insulins in a single formulation, generally administered twice or three-times daily 24,25. These include insulin lispro 75/25 (75% insulin lispro protamine suspension plus 25% insulin lispro), biphasic insulin aspart 70/30 (70% insulin aspart protamine suspension plus 30% insulin aspart) and insulin lispro 50/50 (50% insulin lispro protamine suspension plus 50% insulin lispro). Randomized controlled trials have demonstrated that twice-daily insulin lispro 75/25 or insulin aspart 70/30 and three-times daily insulin lispro 50/50 are significantly superior to basal insulin alone in providing overall and postprandial glycaemic control 26–31. Premixed insulins are, however, associated with an increased risk of daytime hypoglycaemia compared with basal insulin alone. Those who benefit most from premixed insulins compared with basal insulin alone are at a more advanced stage of the disease, necessitating the prandial component of the premixed insulin for postprandial glycaemic control. However, despite the availability of different formulations of premixed insulin, the fixed-ratio nature of premixed formulations make them less flexible and adaptable to the individual's specific needs than a basal-plus strategy 19,24. The remainder of this review will therefore focus on the evidence relating to basal-plus treatment.

### Evidence for the basal-plus strategy for insulin intensification in the management of Type 2 diabetes

The key trials relating to the stepwise intensification of insulin therapy discussed in this review are summarized in Table 1.

**Table 1:** Clinical trials investigating the stepwise addition of bolus insulin to basal insulin

Trial	Duration of randomized treatment period	Trial arms	Population size	HbA_1c_ change (%)	Target HbA_1c_ [mmol/mol (%)]	Proportion achieving target HbA_1c_ (%)	Severe hypoglycaemia rate (events/patient-year)	Weight change, kg
OPAL study 20	24 weeks	Insulin glargine + OADs + insulin glulisine at breakfast	162*	−0.36†	≤ 48 (≤ 6.5)	27.8	0.01 ± 0.15‡	+1.0
		Insulin glargine + OADs + insulin glulisine at main mealtime	154*	−0.31†	≤ 48 (≤ 6.5)	33.8	0.04 ± 0.30§	+0.9
‘Proof of Concept’ study 33	3 months	Insulin glargine + OADs	51*	−0.11†	< 53 (< 7)	8.8	0.2 ± 1.1	−0.4
		Insulin glargine + OADs + insulin glulisine at main mealtime	45*	−0.37†	< 53 (< 7)	22.4	0.0 ± 0.0	+0.7
1.2.3. study 34	24 weeks	Insulin glargine + OADs + once-daily insulin glulisine	115‖	−0.44†	< 53 (< 7)	30	0.28¶	+3.8†
		Insulin glargine + OADs + twice-daily insulin glulisine	113‖	−0.36†	< 53 (< 7)	33	0.89**	+4.1†
		Insulin glargine + OADs + thrice-daily insulin glulisine	115‖	−0.43†	< 53 (< 7)	46	0.64¶	+3.9†
OSIRIS study 35	12 months	Insulin glargine + metformin + thrice-daily insulin glulisine	144*	−0.72 ± 1.25†	NA	NA	NA	+2.03 ± 3.21
		Insulin glargine + metformin + stepwise addition of insulin glulisine (1–3 times daily)††	197*	−0.47 ± 1.05†	NA	NA	NA	+1.30 ± 3.17
		Insulin glargine + metformin + sulphonylurea + stepwise addition of insulin glulisine (1–3 times daily)††	123*	−0.40 ± 1.11†	NA	NA	NA	+1.90 ± 3.38
STEPWise 21	3 × 12 weeks treatment periods	Insulin detemir + OADs + stepwise addition of insulin aspart to largest meal (based on pre-meal glucose values): SimpleSTEP	150	−1.1 ± 1.1	< 53 (< 7)	31	0.04‡‡	+2.7 ± 3.9§§
		Insulin detemir + OADs + stepwise addition of insulin aspart to meal with largest prandial glucose increment (based on post-meal glucose values): ExtraSTEP	146	−1.3 ± 1.2	< 53 (< 7)	27	0.01‡‡	+2.0 ± 3.8§§
ELEONOR 40	24 weeks	Insulin glargine + metformin + once-daily insulin glulisine titrated using SMBG	126‖‖	−0.7 ± 0.06†	< 53 (< 7)	54.8	0.02	+0.4 ± 5.1
		Insulin glargine + metformin + once-daily insulin glulisine titrated using telecare	115‖‖	−0.7 ± 0.06†	< 53 (< 7)	45.2	0.04	+0.4 ± 3.4
All To Target 43	60 weeks	Twice-daily premixed insulin (70/30 protamine-aspart/aspart) + 2–3 OADs	192	−1.8 ± 0.1¶¶	< 53 (< 7)	39	0.2 ± 0.1***	NA
		Insulin glargine + once-daily glulisine + 2–3 OADs	189	−2.1 ± 0.1¶¶	< 53 (< 7)	49	0.1 ± 0.0***	NA
		Insulin glargine + stepwise addition of insulin glulisine + 2–3 OADs	191	−2.2 ± 0.1¶¶	< 53 (< 7)	45	0.2 ± 0.1***	NA

* Per-protocol population.

† Adjusted mean change from baseline.

‡ Safety population *n* = 196.

§ Safety population *n* = 197.

‖ Modified intention-to-treat population.

¶ Safety population *n* = 115.

** Safety population *n* = 113.

†† The first dose of insulin glulisine was added to the meal with the highest postprandial glucose excursion.

‡‡ Major hypoglycaemic episodes—patients unable to treat the episode themselves.

§§ Data expressed as mean ± standard deviation.

‖‖ Intention-to-treat population.

¶¶ Last observation carried forward.

*** Data expressed as adjusted event-rates per patient/year ± standard error.

ELEONOR, Evaluation of Lantus Effect on Optimization of Use of Single Dose Rapid Insulin; NA, data not available; OAD, oral anti-diabetic drug; OPAL, Orals Plus Apidra and Lantus; OSIRIS, Opposing Step-by-Step Insulin Reinforcement to Intensified Strategy; SMBG, self-monitoring of blood glucose.

**FIGURE 3:**
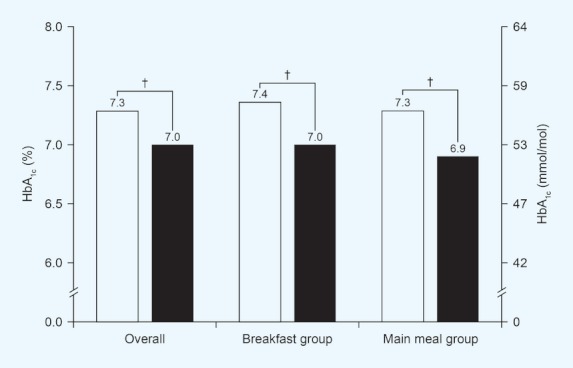
OPAL: improvement in HbA_1c_ in the combined mealtime group and within each mealtime group 20. Individuals received insulin glulisine added to insulin glargine. The ‘main meal’ group also contained individuals whose main meal was breakfast. †*P* < 0.0001 within each group vs. baseline. HbA_1c_ at baseline (□) and endpoint (▪) in the per-protocol analysis set. Predefined margin for equivalence between the breakfast and main mealtime groups at ε = 0.4%, ΔHbA_1C_ (95% CI) = 0.048 (–0.115 to 0.211). Reproduced from Lankisch *et al*. (2008) with permission from John Wiley and Sons Inc.

The OPAL [Orals Plus Apidra® (Sanofi, Paris, France) and Lantus® (Sanofi, Paris, France)] study investigated an early basal plus one prandial insulin injection treatment approach 20,32. Lankisch and colleagues recruited 393 people with Type 2 diabetes inadequately controlled by basal insulin glargine and oral anti-diabetic drugs, who were subsequently randomized to receive a single prandial injection of insulin glulisine for 24 weeks in addition to their existing regimen (insulin glargine plus oral anti-diabetic drugs) 20. The prandial injection was administered either at breakfast or at the individual's main meal (i.e. breakfast, lunch or dinner), as determined by the highest postprandial plasma glucose excursion. The study reported a significant improvement in mean HbA_1c_ levels from baseline, irrespective of whether the meal was ‘breakfast’ or ‘main’ (Fig. 3), with a low and comparable incidence of hypoglycaemia between the two treatment groups.

Additional evidence from the OPAL study showed significant improvements in eight-point glucose profiles following the prandial injection, both at the meal in question and overall. The study also explored the likelihood of individuals achieving HbA_1c_ target recommendations with a single additional prandial insulin injection. A target of ≤ 47.5 mmol/mol (≤ 6.5%) was achieved by 30.7% of individuals, with no statistically significant difference in the number of people meeting this target in the main mealtime group compared with the breakfast group (33.8 vs. 27.8%; *P* = NS), bearing in mind that the individuals randomized to the breakfast group did not overlap with individuals who were randomized to the main meal group, but for whom breakfast was their main meal. Furthermore, in people who had HbA_1c_ > 53 mmol/mol (> 7.0%) at baseline, 44.1% reached a target of < 53 mmol/mol (< 7%) at endpoint (52.2 and 36.5% for main mealtime and breakfast groups, respectively; *P* = 0.028) 20.

A later OPAL subanalysis suggested that the improvements in glycaemic control observed in the total study population were also observed in individuals already close to metabolic target [HbA_1c_ 53.0–58.5 mmol/mol (7.0–7.5%)] 32. Out of these individuals, those who received prandial insulin at their main meal experienced more of an effect than those receiving prandial insulin at breakfast [66 and 52% in the main-meal and breakfast groups, respectively, achieving an HbA_1c_ ≤ 53 mmol/mol (≤ 7.0%)] 32. Overall, results from the OPAL study demonstrated that the introduction of a single bolus dose of prandial insulin, added to basal insulin and oral anti-diabetic drugs, had the potential to offer a simple and effective means of intensifying insulin therapy in people with Type 2 diabetes.

### Proving the concept

**FIGURE 4:**
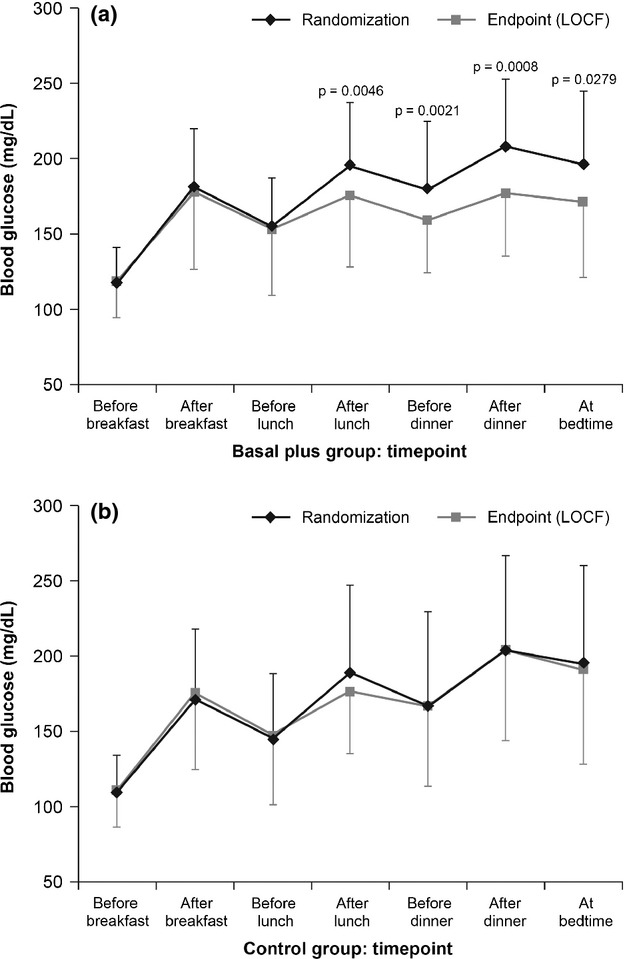
Proof-of-concept: seven-point self-monitored blood glucose profiles in the bolus (a) and control (b) groups 33. Individuals received insulin glulisine added to insulin glargine. Blood glucose was monitored before breakfast (fasting), lunch and dinner, 2 h after each meal and before bedtime. Results are means ± standard deviation; LOCF, last observation carried forward. Reproduced from Owens *et al*. (2011) with permission from John Wiley and Sons Inc.

A limitation of the OPAL study was that existing basal insulin therapy was not optimized before the addition of prandial insulin. Consequently a 6-month, proof-of-concept study was subsequently designed to further explore this gradual approach to insulin intensification 33. Eligible individuals initially underwent a 3-month run-in period on insulin glargine, titrated to optimize fasting blood glucose control, after which those whose HbA_1c_ was > 53 mmol/mol (> 7.0%) were randomized to either continue with basal insulin glargine (‘basal-only’; *n* = 57) or add a single dose of insulin glulisine immediately before their main meal (‘basal-plus’; *n* = 49). After 3 months, the proportion of individuals attaining an HbA_1c_ target of < 53 mmol/mol (< 7.0%) was significantly higher for basal-plus vs. basal-only treatment (22.4 vs. 8.8%; *P* < 0.05). In addition, the reduction in HbA_1c_ was significantly greater using basal-plus vs. basal-only treatment (–0.37 vs. –0.11%; *P* = 0.029). Rates of hypoglycaemia and weight change were comparable between the two treatment groups. A subanalysis of people in the basal-plus group by prandial injection time (i.e. before breakfast, lunch or dinner) showed similar numbers achieving target, with no difference in endpoint HbA_1c_. The timing of the insulin glulisine injection did not affect safety or weight gain. Seven-point, self-monitored blood glucose profiles also showed significant improvements between randomization and endpoint in the basal-plus treatment group (Fig. 4a), but not in the basal-only group (Fig. 4b).

The results from this study indicated that, compared with basal insulin alone, advancing from a basal-only to a basal-plus regimen is an effective and safe option in people with Type 2 diabetes 33.

### Intensifying prandial insulin treatment

**FIGURE 5:**
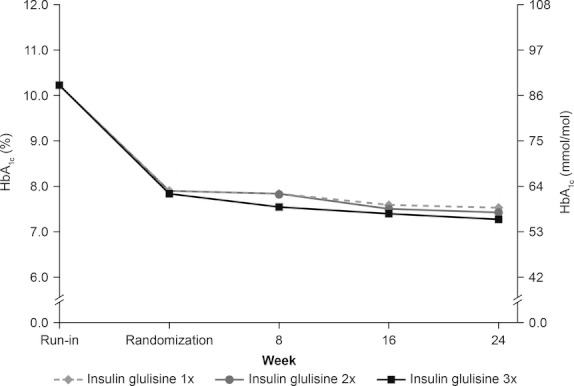
1-2-3 study: evolution of HbA_1c_ in the randomized population 34. Individuals received insulin glulisine added to insulin glargine. Reproduced from Davidson *et al*. (2011) with permission from the American Association of Clinical Endocrinologists.

The efficacy and safety of adding a single dose of prandial insulin to an existing basal regimen has been demonstrated 32, but some individuals are still unable to reach recommended HbA_1c_ targets. Therefore, the next step is further intensification of prandial insulin therapy to two daily injections before instigating a full basal–bolus regimen. In the 1-2-3 study, individuals who had previously received two or three oral anti-diabetic drugs for at least 3 months entered a 14-week insulin glargine run-in period, after which those whose HbA_1c_ was > 53 mmol/mol (> 7.0%) were randomized to receive insulin glulisine once (*n* = 115), twice (*n* = 113) or three times (*n* = 115) daily for 24 weeks. Dose adjustments were made weekly based on whether preprandial blood glucose values were low or high 34. After 24 weeks, reductions in HbA_1c_ achieved with once- and twice-daily insulin glulisine were shown to be non-inferior to the reduction achieved with three-times-daily administration (reduction from randomization: –0.44, –0.36 vs. –0.43%, respectively; Fig. 5). The proportion of individuals achieving an HbA_1c_ target of < 53 mmol/mol (< 7%) after 24 weeks of once-, twice- or three-times-daily insulin glulisine treatment was 30, 33 and 46%, respectively. Weight gain was reportedly low and similar across all three treatment groups, whereas the rates of confirmed symptomatic hypoglycaemia were 12.2, 12.9 and 17.1 events/person-year in the insulin glulisine once-, twice- and three-times-daily groups, respectively. The mean insulin dose (glargine + glulisine) was comparable across the insulin glulisine once-, twice- and three-times-daily groups at the study's end (1.05, 1.21 and 1.44 U/kg, respectively). Ultimately, the 1-2-3 study demonstrated that once- and twice-daily insulin glulisine were non-inferior to insulin glulisine administered three times daily 34.

Unlike the 1-2-3 study, which reported findings across individual treatment groups receiving different numbers of injections, the design of the OSIRIS (Opposing Step-by-step Insulin Reinforcement to Intensified Strategy) study allowed for stepwise intensification from one to three doses of prandial insulin within an individual treatment group 35. People with Type 2 diabetes poorly controlled by basal insulin and oral anti-diabetic drugs (*n* = 811) were switched to insulin glargine for 6 months, while continuing their oral anti-diabetic drugs. After completing this run-in period, 476 people with HbA_1c_ > 53 mmol/mol (> 7%) and fasting plasma glucose < 6.7 mmol/l were randomized to either a basal–bolus regimen or one of two stepwise regimens for 12 months. The basal–bolus regimen comprised insulin glargine plus metformin plus up to three-times-daily prandial insulin glulisine. The stepwise regimens both comprised insulin glargine plus metformin plus one- to three-times-daily insulin glulisine, but differed in that one arm also received a sulphonylurea. Insulin glulisine was initially administered at the main meal (highest postprandial glucose excursion), with two further doses introduced at months 4 and 8 if HbA_1c_ remained > 53 mmol/mol (> 7%). At the study's end, stepwise insulin glulisine treatment showed efficacy that was similar to that achieved with the basal–bolus approach. Furthermore, the incidence of symptomatic hypoglycaemia was low and comparable between treatment groups 35. Thus, the OSIRIS study confirmed that adding one injection of insulin glulisine to insulin glargine had similar efficacy and safety to a full basal–bolus regimen in Type 2 diabetes management.

### When to initiate prandial insulin—simplifying meal choice

Several studies have investigated the stepwise intensification of insulin therapy, particularly in terms of comparing a basal plus one prandial insulin vs. a full basal–bolus strategy 32–35. The majority of these studies have selected an initiation point for prandial insulin administration based on the HbA_1c_ level, and/or self-monitored fasting blood glucose and degree of glycaemic excursion after mealtimes. However, a recent study with insulin aspart and insulin detemir as the prandial and basal insulins, respectively, challenged this notion by investigating two different strategies for determining the ‘main meal’, i.e. the starting point for basal-plus initiation 21. The 48-week, randomized, open-label STEP-Wise™ study, conducted in individuals with inadequately controlled Type 2 diabetes on basal insulin and oral anti-diabetic drugs, used two different methods to determine the meal to be targeted for prandial injection 21. Insulin aspart was initially added to either the meal considered the largest by the individual (titration based on pre-meal glucose values; SimpleSTEP) or to the meal with the largest glucose excursion (titration based on post-meal values; ExtraSTEP). Following a 12-week run-in period whereby insulin detemir was optimized, 296 people with HbA_1c_ ≥ 53 mmol/mol (≥ 7.0%) were randomized to either the SimpleSTEP or the ExtraSTEP strategy before receiving insulin aspart at the dedicated meal, with treatment intensification from one to three injections occurring at fixed time points every 12 weeks if HbA_1c_ remained ≥ 53 mmol/mol (≥ 7.0%).

**FIGURE 6:**
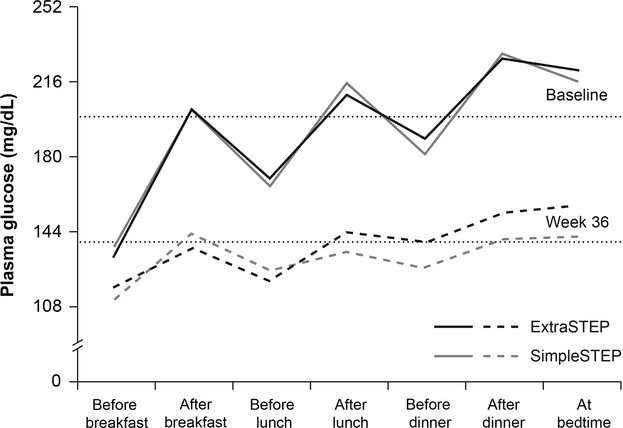
STEP-Wise™ Study: seven-point self-monitored blood glucose profiles at baseline and study end in the SimpleSTEP and ExtraSTEP treatment groups 21. Individuals received insulin aspart added to insulin detemir. Mean seven-point plasma glucose profiles at baseline (solid lines) and after 36 weeks of treatment (heavy dashed lines). Light dashed lines indicate reference points for 7.99 mmol/l (144 mg/dl) and 10.99 mmol/l (198 mg/dl). Reproduced from Meneghini *et al*. (2011) with permission from the American Association of Clinical Endocrinologists.

Both the SimpleSTEP and ExtraSTEP treatment strategies improved HbA_1c_ reductions to a similar degree, with a similar proportion of people reaching an HbA_1c_ target of < 53 mmol/mol (< 7%) at 36 weeks (31 and 27%, respectively; *P* = 0.74). Similar results were also reported between the two groups for fasting plasma glucose and reductions in mean seven-point glucose profiles, although the pattern of change for postprandial glucose excursions was slightly different between the two treatment groups (Fig. 6). Weight gain, adverse event rate and number of hypoglycaemic episodes were also low and similar between the SimpleSTEP and ExtraSTEP strategies, although, as might be expected, hypoglycaemia frequency increased with increasing number of prandial injections, from one to three, in both groups (SimpleSTEP: 6.2 to 14.7 events/year; ExtraSTEP: 5.6 to 13.0 events/year).

Findings from the STEP-Wise study showed that different methods of choosing the meal to be covered by prandial insulin did not affect efficacy and safety outcomes. Furthermore, STEP-Wise reported overall improvements in HbA_1c_ with sequential intensification of prandial insulin (approximately 75% of people required three prandial injections by study end), although, as with the OSIRIS study, no within-treatment-group efficacy analyses seem to have been published so far. Of the two strategies tested, SimpleSTEP has the potential to be more readily acceptable, because of the ‘simpler’ selection of the ‘main meal’, compared with the ExtraSTEP strategy.

Findings from the OPAL study also showed similar benefits in efficacy outcomes, whether individuals received a single prandial injection at breakfast or at their main meal 20. Further studies are needed to verify whether or not ‘main-meal identification’ is critical or whether a breakfast injection is adequate for all individuals requiring intensification of their insulin treatment.

### How to adjust prandial insulin—simplifying titration

Adjustment of insulin doses is an important factor in maintaining optimized glycaemic control while minimizing the risk of hypoglycaemia. This has been shown in individuals with Type 1 diabetes to be best performed by carbohydrate counting, the gold standard for glycaemic control in this population 36,37. However, using this method to adjust the insulin dose can be very complicated and time-consuming 38,39. A multi-centre, randomized, parallel-group study by Bergenstal *et al*. compared the use of a simple weekly dose-adjustment algorithm for prandial insulin glulisine with the more complex carbohydrate-counting algorithm in 273 persons with Type 2 diabetes undergoing full basal–bolus therapy 40. Both groups received basal insulin glargine titrated to target fasting blood glucose levels, based on the mean fasting blood glucose level of the previous 3 days of self-monitoring of blood glucose. Prandial insulin was also adjusted weekly based on pre-lunch, pre-dinner and bedtime self-monitoring of blood glucose results from the previous week, with the simple algorithm providing a set dose of insulin to inject preprandially, and the carbohydrate-counting algorithm providing a dose per amount of carbohydrate in the meal. The study found that the simple algorithm was as effective as the carbohydrate-counting system, in terms of both efficacy and safety, with no significant difference in the mean change from baseline in HbA_1c_. The findings suggest that the complexity introduced when prandial insulin is added, both for the individual and the physician, can be minimized by the use of a simple dosing algorithm, thereby removing one of the potential barriers to the introduction and optimization of prandial insulin.

In the ELEONOR study, a 24-week trial conducted in 224 insulin-naïve people with poorly controlled Type 2 diabetes, the efficacy and safety of a basal-plus treatment strategy, comprising insulin glargine plus a single dose of insulin glulisine at the meal with the highest blood glucose excursion, were shown to be unaffected by two different methods of monitoring for dose adjustment, i.e. Telecare or self-monitoring of blood glucose 41. ELEONOR indicates that people with Type 2 diabetes adapt well to a basal-plus strategy using a simple dosing algorithm without undue additional burden on the healthcare professional. The study also confirms that a basal-plus regimen is associated with significant improvements in glycaemic control, with limited weight gain and a low rate of hypoglycaemic events. The ELEONOR study also demonstrated that the improvement in glycaemic control had a positive effect on physical and psychological well-being and treatment satisfaction, which was greater with the basal-plus regimen 42.

### Basal-plus vs. premixed insulin

The All to Target study in Type 2 diabetes was a 60-week, randomized, open-label study that compared (1) insulin glargine plus one injection of insulin glulisine, (2) insulin glargine plus up to three injections of insulin glulisine and (3) two injections of premixed insulin (biphasic insulin aspart 70/30) 43. Initial results showed that both insulin glargine/insulin glulisine regimens lowered blood glucose levels compared with premixed insulin, but with more individuals reaching target HbA_1c_ and with less hypoglycaemia. While non-inferiority of the basal-plus (≤ 1 prandial injection) vs. premixed insulin was demonstrated, superiority of basal-plus (≤ 3 prandial injections) vs. premixed insulin was not achieved.

A subgroup analysis of the All to Target study compared outcomes in individuals who received insulin glargine alone or in combination with one, two or three daily insulin glulisine doses 44. Individuals who received insulin glargine alone or in combination with one insulin glulisine dose comprised 62% of the population analysed. Those who received insulin glargine in combination with three plus doses of insulin glulisine (*n* = 191; 18% of the overall population) had significantly higher mean baseline HbA_1c_ levels than the other subgroups (*P* < 0.05 for all comparisons). Furthermore, after 60 weeks of treatment, mean HbA_1C_ levels were still significantly higher in the subgroup of individuals who received three or more doses of insulin glulisine than in those who received insulin glargine alone or in combination with one or two doses of insulin glulisine (8.3 vs. 6.8, 6.9 and 7.3%, respectively; *P* < 0.001 for all comparisons). The findings indicate that, in the early stages of insulin therapy, most individuals (approximately two-thirds) achieve favourable glycaemic control with insulin glargine alone or in combination with one prandial dose of insulin glulisine; however, there seems to be a subgroup of individuals whose glycaemic control remains suboptimal, even when a full basal–bolus regimen is used 44.

### When nothing but a full basal–bolus regimen will do

If glycaemic control continues to deteriorate, an individual will eventually require a full basal–bolus regimen. In light of this relatively demanding regimen, a physician may consider whether or not it would be beneficial for a person with Type 2 diabetes in this position to remain on a basal–bolus regimen, switch to a twice- or thrice-daily premixed insulin or go on to continuous subcutaneous insulin infusion therapy. The comparative benefits of basal–bolus and premixed insulin strategies were explored in the Glulisine in Combination with Insulin Glargine in an Intensified Insulin Regimen (GINGER) and the 4-T studies, as well as a trial conducted by Rosenstock *et al*. that compared lispro mix with basal–bolus insulin glargine and lispro 17,18,45–47.

The 24-week, randomized study by Rosenstock *et al*. was designed to compare basal–bolus therapy (insulin glargine plus prandial insulin lispro; *n* = 187) with thrice-daily premixed prandial therapy (lispro mix 50/50; *n* = 187) in individuals inadequately controlled [HbA_1c_ ≥ 58 mmol/mol (≥ 7.5%) and ≤ 108 mmol/mol (≤ 12%)] with insulin glargine and oral anti-diabetic drugs 46. Both treatment arms achieved similar reductions in HbA_1c_ by the end of the randomized treatment period. The difference in the change in HbA_1c_ between the two treatment regimens was 0.22% (basal–bolus –2.09%; premixed insulin –1.87%). However, a greater number of people in the basal–bolus arm achieved the target of HbA_1c_ < 53 mmol/mol (< 7%; 69 and 54% for basal–bolus and premixed insulin, respectively; *P* < 0.05). No significant differences were seen in the rate of hypoglycaemia or amount of weight gain in this study.

The GINGER study compared the safety and efficacy of basal–bolus insulin (insulin glargine plus insulin glulisine with meals) vs. twice-daily premixed insulin (either Neutral Protamine Hagedorn [NPH]/regular human insulin or biphasic insulin aspart 70/30) in 310 people with long-standing Type 2 diabetes 45,47. The study reported a significantly greater mean decrease in HbA_1c_ (–1.31 vs. –0.80%; *P* = 0.0001), smoother eight-point self-monitored blood glucose profiles, and a significantly greater proportion of people reaching an HbA_1c_ target of ≤ 53 mmol/mol (≤ 7.0%; 46.6 vs. 27.9%; *P* = 0.0004) in the basal–bolus group compared with the premixed group, with no difference in the rate of hypoglycaemia. Mean daytime glycaemia and postprandial glucose were lower with basal–bolus therapy (*P* < 0.0001), while the daily insulin dose was similar for both groups (∼90 units). A small but significant weight gain was observed with the basal–bolus vs. premixed insulin regimen (*P* = 0.0073); however, this was not considered to be clinically relevant. An additional analysis from the GINGER study showed that, even relative to endpoint HbA_1c_, the rate of overall hypoglycaemia was 24.5% lower in the basal–bolus group than in the premixed group (*P* = 0.1211), with a 43.3% lower rate of overall hypoglycaemia compared with the premixed subgroup receiving NPH/aspart (*P* = 0.0196) 45,47.

The 4-T study also provided insight into the benefits of basal–bolus therapy over premixed insulin. Although participants in the 4-T study initiated insulin treatment with basal (once- or twice-daily insulin detemir), prandial (three-times-daily insulin aspart) or premixed (biphasic insulin aspart) regimens, treatment could be intensified if HbA_1c_ levels increased according to predefined criteria. In the case of twice-daily premixed insulin, insulin aspart was added at lunchtime, whilst the basal and prandial arms were switched to a full basal–bolus regimen 17,18. At the end of the third year of the study, 67.7, 73.6 and 81.6% of people in the premixed, prandial and basal insulin groups, respectively, were receiving a second type of insulin. Approximately two-thirds of the people who received full basal–bolus therapy achieved and maintained the target HbA_1c_ of 53 mmol/mol (7.0%). Similar mean HbA_1c_ and daily insulin doses were observed for the groups initially receiving basal or prandial insulin, although those initiated on basal insulin experienced less weight gain and fewer hypoglycaemic events 17.

Findings from the GINGER and 4-T studies demonstrate that a basal–bolus regimen offers improved glycaemic control, better target achievement and lower rates of hypoglycaemia than premixed insulin therapy 17,18,47. The study by Rosenstock *et al*. found that a full basal–bolus regimen allowed more people to achieve glycaemic targets than a premixed regimen, but that the two regimens were equivalent in terms of overall glycaemic control, weight gain and rate of hypoglycaemia 46. In addition, the 4-T study indicated that individuals started on a basal insulin regimen are more likely to reach their target HbA_1c_ levels, with fewer hypoglycaemic episodes and less weight gain, when they subsequently need to progress to a full basal–bolus regimen 17. The basal–bolus approach also permits flexibility in dosing adjustment compared with the premixed option 19.

### Implications for clinical practice

**FIGURE 7:**
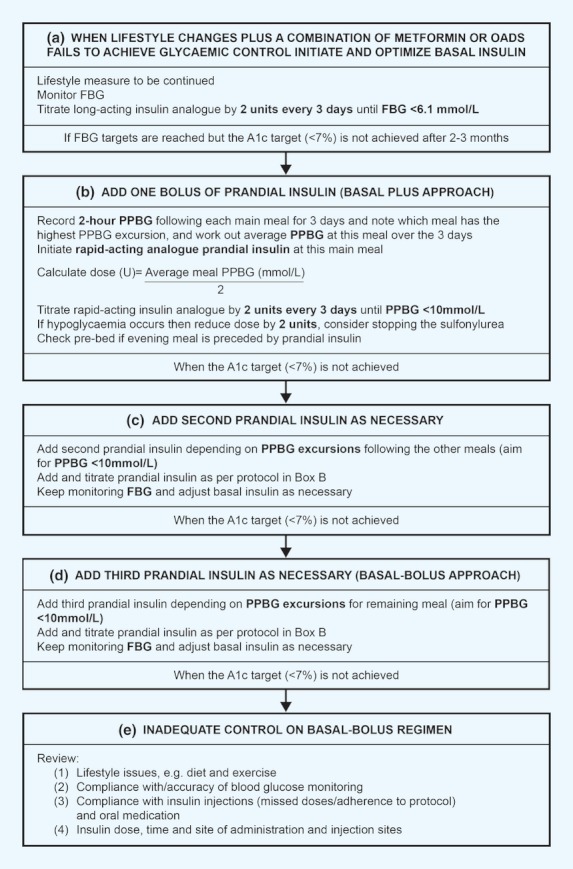
A simple treatment algorithm based on practical, clinical experience for the initiation and titration of rapid-acting insulin 48. FBG, fasting blood glucose; PPBG, postprandial blood glucose; U, international units of insulin. Reproduced from Owens *et al*. (2009) with permission from Wiley-Blackwell.

Current evidence demonstrates that the basal-plus strategy provides a graduated advancement of insulin therapy based on each individual's requirements. This approach has been shown to be effective and accompanied by low rates of hypoglycaemia and minimal weight gain. The basal-plus approach to insulin intensification in persons with Type 2 diabetes seems to be a promising alternative to the current options of either twice-daily premixed insulin or a full basal–bolus strategy, thereby providing a viable intermediate step between basal only and basal–bolus strategies, as recommended in the latest version of the ADA/EASD consensus guidelines (Fig. 2) 19. The basal-plus strategy is potentially suitable for a large number of individuals with Type 2 diabetes, and may delay their progression to a full insulin replacement regimen, especially when the initiation of insulin therapy is not delayed. Figure 7 shows a potential framework for the initiation and titration of rapid-acting insulin as part of a basal-plus strategy that is both practical and realistic.

## Conclusions

Several studies have demonstrated the clinical potential of a stepwise intensification of prandial insulin, building on an existing basal insulin regimen, for the management of Type 2 diabetes. This ‘basal-plus’ strategy could increase the therapeutic options available to the person with diabetes, permitting further individualization of their insulin therapy, which may translate into increased clinical and functional benefits. The meal-driven, graduated progression of insulin therapy in this way, rather than immediately embarking on a full basal–bolus regimen or introducing premixed insulin preparations, may be more relevant and acceptable to a large number of persons with Type 2 diabetes and their physicians alike. However, the clinical situation may demand an earlier introduction of a full basal–bolus regimen in certain individuals. Whilst the use of basal–bolus insulin therapy confers certain benefits over and above premixed insulin-based regimens, a greater commitment by the individual concerned and their carers is required in order to maximize these advantages.
